# Cases Cured by Dr. Dahlke, of Berlin

**Published:** 1893-02

**Authors:** 


### CASES CURED.

By Dr. Dahlke, Berlin, Germany.

(From the *Zeitschrift des Berlin Vereines hom. Aerzte.*)

Translated by A. McNeil, M. D., San Francisco.

A pale, thin man in the forties, complains of attacks of vertigo, which are sometimes accompanied, by vomiting. He has, also, a confused feeling of fullness in the head and great bodily weakness. Gelsem.$^{2}$, three globules three times a day.

After eight days the same condition. A second examination shows that the attacks of vertigo come more frequently in the open air, and in the very moment of lying down, if his pillow is low, consequently he must lie with his head high.

Sepia$^{9}$, three globules, one a day, was followed by immediate improvement and cure. The Sepia symptom " vertigo, only when walking in the open air " is well known. A sympton of vertigo, when lying down, does not exist to my knowledge (in Sepia). Symptom 99 of Hahnemann's proving, is " staggering vertigo every afternoon, from four to six, when sitting and walking." Apis has vertigo worse when sitting, very severe when lying, and closing the eyes. Lachesis has vertigo worse when sitting, and vertigo on sitting. It has cured vertigo on closing the eyes, and vertigo after lying down (Hering, *Werkungen des Schlangengiftes*). According to Farrington, Theridion, and perhaps Moschus also, have the same symptom.

A man of forty-nine, subject to temporary attacks of gout, consulted me December 23d, for the following complaints : A headache begins every morning about eight o'clock, in the middle of the forehead, which reaches its maximum between ten and eleven A. M., and then decreases till evening, when it disappears entirely. It is accompanied by great irritability, sensitiveness of the skin of the forehead, and of the ridge of the nose to pressure ; feeling of dryness in the nose.

Natrum-mur.$^{11}$, three globules night and morning.

On the next day the pain was almost entirely gone, and did not return as long as he was under my treatment--*i. e.*, till January 22d.

I gave Natrum-mur. in the first place on account of the time of the pains. They began in the morning, increased till toward noon, and then gradually decreased. With Spigelia, as is well known, the headache follows the sun. Farrington also mentions Gelsemium and Glonoinum as having this symptom. However, it appears as if with the two latter remedies that it is the intensity of the heat of the sun which causes the headache. Glon. is a chief remedy for heat-stroke, and Gelsemium is particularly indicated in complaints which arise after hot, damp weather. Kali-bichrom. and Verbaseum have pains with the same typical course. The time of aggravation—between ten and eleven A. M.—is characteristic of Natrum-mur. It has an intermittent fever, with chill, at this time. There is hardly a time of aggravation excepting the general ones of morning, evening, and night, so frequent in our *materia medica*, as this of from ten to eleven A. M. Sulphur is the best known of them. Some of the others are Argentum-nit., Arsenicum, Iodium, Ipecac., Natrum-carb., Phosphorous, Stannum, and Zinc. What decided my choice for Natrum-mur. was the feeling of dryness in the nose. Salt has this sensation in different mucous membranes. The typical Natrum-mur. headache is accompanied by dryness of the mouth. If I am not mistaken greatly, I have read a proving of Crotalus containing this symptom : " Headache, with dry mouth and cold feet." [Neither the *Guiding Symptoms* nor *Allen's Enclycopædia* has it—Traus.] Natrum-mur. has the tongue feels dry, but is not (to touch). Argent-nit. has it also. For many complaints with exterior dryness of the mouth, Nux-moschata is suitable. A very extensive feeling of dryness belongs to all the carbon preparations. One patient had, besides, a remarkable coldness of the hands, which was formerly worse than now, but still necessitated his wearing gloves at night during sleep. This is incidentally a Natrum-mur. symptom, which I had not discovered at the time I prescribed for his headache. I have found the same symptoms in Petroleum. I therefore add to the Natrum-mur. symptoms, " Headache with dryness of the mouth."

We endeavor in selecting the remedy to discover a drug which has symptoms that agree the closest with the morbid picture.

A medicine is so much the better indicated for a certain case of disease if there are the greatest possible number of symptoms in common. That is the general rule. Now it too often happens to all of us that a remedy does not help notwithstanding the greatest possible agreement of the symptoms. One often accounts for that by speaking of it as a mere covering of symptoms, and so the failure is attributed to a superficial way of prescribing. At all events, we cannot succeed in curing by the number and quantity of the symptoms alone in common of the case and the drug. It therefore follows that we must discriminate in the quality of the symptoms. This has led to the principle of "guiding symptoms." It is of great importance. The guiding symptom is, as it were, a guide-post, certainly it is one on which several terminal points are mentioned.

It is also true that there are other aids to simplify and make more accurate the choice of *the* remedy. One of these is ætiology, the value of which Helbig successfully demonstrated. Strictly speaking, when we utilize this in prescribing, we go beyond the fundamental principles of Homœopathy if not those of Hahnemann. [The Doctor is in error, for see the *Materia Medica Pura*, how he prescribed Aconite for the bad effects of fear and fright, China for excessive loss of fluids, Ignatia for the results of chagrin, etc.—Trans.] One might say that Hahnemann on this point was not homœopathic enough. In the meantime, when a thousand-fold experience has demonstrated an inner relationship between certain remedies and certain causes of disease, and moreover, as Helbig has clearly shown that aggravations and ameliorations of certain complaints by motion, rest, time of day, touch, and the like are nothing else than causes of disease, so that an ætiological point of view is indispensable. Considering these very convincing grounds, there are but few indeed, whether or not believers in the psoric theory, who will deny that ætiology is a constituent part of Homœopathy.

In reference to these "guiding symptoms," theoretically all symptoms have an equal value. If we discriminate we find that those symptoms that are more accessible and which we can expect to discover most frequently at the bedside should be preferred. It is not exactly, but approximately true to say that we succeed in the opposite way in discovering the guiding symptoms of a remedy. They are for the most part rare symptoms which have something striking about them and are therefore usually easily recognized. Sometimes it is a single symptom such as a "cold feeling in the heart," "heaviness in the rectum," "intermittent pulse," "splitting pains," etc. Sometimes it is symptoms standing in a certain intimate connection—symptom-combinations which, if I may be permitted to use the comparison, differ from the multitude of symptoms occurring together as a chemical compound does from a mere mixture. For example, there are the three characteristic symptoms of Sulphur: Heat in the head, cold feet, and the hungry feeling at eleven A. M. Such symptom-combinations have, in my opinion, a high value in selecting the remedy, and cannot be outweighed by any number of other symptoms which accidentally stand together. A single symptom is like a point. In one point an infinite number of mathematical figures may fall together without otherwise coinciding. Two points united together form a line and assume a definite direction. Finally three or more united together constitute an inclosed figure. I pray you not to confound this with Hering's three points, which are elaborated in his final remarks on Kalmia.

I find it difficult to tell all I have to say on the subject and to make everything clear. I therefore again take refuge in a comparison. In chemistry there are substances which are identical or almost so in their formula and are not all alike in their nature or composition. As the relationship between two substances rests not alone on the presence of like elements and quantities, but on the grouping, structure, and inner composition of the individual parts, so is the relationship between the morbid and the medicinal picture.

My idea is not that the nature of such combinations of symptoms demand that the very same symptoms should occur in the case in point and the remedy but that an inner relationship should exist between them, a direct depending of the one upon the other, that one should be conditioned upon the remaining ones and they upon that one.

I have found an excellent expression in Darwin for this inner relationship between phenomena, and the regular sequence of their occurring with each other or in succession to each other. He calls it the principle of correlation, and mentions as one example, among others, the coincidence of a strong muscular development and strongly projecting supra-orbital protuberances. I must again expose myself to the danger of being reproached with being unscientific by quoting Cervantes. When Don Quixote heard of the mole on Dulcina's right cheek, he said, "Judging by this mole, as there is a relationship between the face and the other parts of the body, Dulcina must have a mole on the fleshy part of her thigh of that side on which the mole on her face lies."

The correlative symptoms, as we may now call them, exist either in the same locality or follow each other in succession.

As far as I know, Hering was the first one who devoted attention to this succession of symptoms in order of time. He was led to do so, as he states, by the peculiar form of the Sabadilla headache. Records of cases are at every one's command whose characteristic symptoms depend more on time than on space. It is clear that the law of the *similia* gains thereby greater depths. We go, if I may so express it, from the plane trigonometrical treatment of the symptom-complex to the stereometrical; from the superficial to the cubical measurement.

Necessarily by so doing the difficulty of selecting the remedy increases, but just as necessarily its certainty becomes greater. Every progress must be obtained by a corresponding amount of labor. Both stand in an unchangeable relationship to each other. Lawrence Sterne's northwest passage in the intellectual world does not exist. On the contrary, all the pains taken for any subject will reappear in some form. The law of the correlation of forces redounds to our benefit.

